# A Phase I Study of Pelabresib (CPI-0610), a Small-Molecule Inhibitor of BET Proteins, in Patients with Relapsed or Refractory Lymphoma

**DOI:** 10.1158/2767-9764.CRC-22-0060

**Published:** 2022-08-11

**Authors:** Kristie A. Blum, Jeffrey G. Supko, Michael B. Maris, Ian W. Flinn, Andre Goy, Anas Younes, Suresh Bobba, Adrian M. Senderowicz, Sergey Efuni, Ronda Rippley, Gozde Colak, Patrick Trojer, Jeremy S. Abramson

**Affiliations:** 1 Emory University School of Medicine, Atlanta, Georgia.; 2 Massachusetts General Hospital, Boston, Massachusetts.; 3 Colorado Blood Cancer Institute, Denver, Colorado.; 4 Sarah Cannon Research Institute and Tennessee Oncology PLLC, Nashville, Tennessee.; 5 John Theurer Cancer Center, Hackensack University Medical Center, Hackensack, New Jersey.; 6 Memorial Sloan Kettering Cancer Center, New York City, New York.; 7 Constellation Pharmaceuticals (a Morphosys Company), Boston, Massachusetts.; 8 Constellation Pharmaceuticals (a Morphosys Company), Boston, Massachusetts.

## Abstract

**Purpose:**

NF-κB, a transcription factor essential for inflammatory responses, is constitutively activated in many lymphomas. In preclinical studies, pelabresib (CPI-0610), an investigational (BET) bromodomain inhibitor, downregulated NF-κB signaling and demonstrated antitumor activity *in vitro*. Here we report the safety, pharmacokinetics, pharmacodynamics, and preliminary clinical activity from the first-in-human phase I study of pelabresib in patients with relapsed/refractory lymphomas (NCT01949883).

**Experimental Design:**

Sixty-four patients with relapsed/refractory lymphoma (median of 4 prior lines of therapy) were treated with either capsule (6, 12, 24, 48, 80, 120, 170, 230, 300 mg) or tablet (125, 225 mg) doses of pelabresib orally once daily on a 14 days on, 7 days off schedule.

**Results:**

The MTD was determined as the 225 mg tablet daily. The most frequent adverse events were fatigue, nausea, and decreased appetite. Thrombocytopenia, a class effect for all BET inhibitors, was dose-dependent, reversible, and noncumulative. Pelabresib exhibited dose-proportional increases in systemic exposure, rapid absorption, and a half-life of approximately 15 hours (supporting once daily dosing). The bioavailability of the tablet formulation was 60% greater than the capsules. Pelabresib suppressed *IL8* and *CCR1* mRNA at doses above 120 and 170 mg, respectively. Four patients (6.2%) had an objective response (2 complete response and 2 partial response) and 5 patients had prolonged stable disease.

**Conclusions/Discussion:**

Pelabresib is capable of BET target gene suppression in an exposure-dependent manner with an acceptable safety profile leading to the recommended phase II dose of the 125 mg tablet once daily.

**Significance:**

BET proteins inhibition can potentially modify the pathogenic pathways which contribute to many diseases including malignancies. Pelabresib (CPI-0610), a potent and selective small molecule BET proteins inhibitor, has a MTD of 225 mg once daily for 14 days with a 7-day break, clear pharmacokinetic/pharmacodynamic relationship, and manageable clinical safety profile. These findings are part of the foundation for the ongoing pivotal study of pelabresib in patients with myelofibrosis.

## Introduction

Bromodomain and extraterminal domain (BET) proteins include a family of four related proteins (BRD2, BRD3, BRD4, and BRDT), each containing two tandem amino-terminal bromodomains (BD1 and BD2) that regulate the expression of an array of genes (1, 2). BET inhibition has the potential to modify multiple critical components of lymphomagenesis, including cell proliferation, cell fate, and survival (3, 4). BET inhibition can attenuate Nuclear Factor-κB (NF-κB) pathway and IκB kinase signaling resulting in rapid reversal of expression of specific genes including B-cell lymphoma 2, *IL6*, *IL8*, and *IL10* (5). Modulation of C-C motif chemokine receptor 1 (CCR1) by BET inhibition has been observed in a number of human hematologic and solid tumor cell lines, xenograft models, and *ex vivo* treated human peripheral blood mononuclear cells (6, 7). In addition, 50% suppression of *CCR1* expression has been used as a pharmacodynamic (PD) marker of BET inhibition in clinical trials, where it was associated with clinical response in patients with relapsed or refractory lymphoma (8).

While BET proteins have a broad distribution across the genome and BET inhibition has widespread effects on gene expression, interference with BET protein binding to chromatin using small-molecule inhibitors of their bromodomains has more circumscribed and selective effects. Three groups (9–11) have independently confirmed that suppression of *MYC* transcription plays a dominant role in mediating the phenotypic effects of BET inhibition. Maximal inhibition of transcription is achieved within approximately 4 hours, and is accompanied by decreases in the level of *MYC* protein.

Pelabresib (CPI-0610) is a synthetic, small-molecule inhibitor of the tandem amino-terminal bromodomains (BD1 and BD2) of BET proteins (12) currently in clinical development.

In preclinical studies, pelabresib treatment results in downregulation of NF-κB signaling activity, accompanied by loss of viability in ABC- diffuse large B-cell lymphoma (DLBCL) and Burkitt lymphoma (BL) cell lines. A study conducted in immunodeficient mice bearing subcutaneous xenografts of Raji Burkitt lymphoma demonstrated that pelabresib inhibits the expression of *MYC* in tumor tissue in a dose-dependent manner. Rearrangement of the *MYC* proto-oncogene serves as a defining feature for BL, but it also occurs in subsets of DLBCL and high-grade B-cell lymphomas. Following administration of a single 30 mg/kg oral dose of pelabresib, maximum inhibition of *MYC* expression (75%) was achieved 4 hours postdosing (the earliest postdosing time point assessed). Suppression of *MYC* expression persisted at 8 hours postdosing, but by 12 hours, *MYC* expression had returned to its baseline value, consistent with pelabresib's rapid clearance in mice (11).

Here we present the safety, pharmacokinetics and pharmacodynamics (PK/PD), antitumor activity results, and maximum tolerated dose (MTD) from a first-in-human phase 1 study in patients with relapsed or refractory lymphoma to support the recommended phase 2 dose (RP2D) and dosing schedule of pelabresib.

## Materials and Methods

### Study Design

Study 0610–01 was a first-in-human phase 1, multi-center, open-label, dose-escalation study of pelabresib (CPI-0610) in patients with relapsed or refractory lymphoma (NCT01949883).

Eligible patients for treatment with pelabresib were adults with relapsed non-Hodgkin or Hodgkin lymphoma after at least one prior therapy or who were not eligible for high-dose chemotherapy and autologous stem cell transplantation or had refused high-dose chemotherapy and autologous stem cell transplantation. Patients were required to have platelet count ≥75 × 10^9^/L, absolute neutrophil count ≥1.0 × 10^9^/L, and Eastern Cooperative Oncology Group performance status of 2 or less.

Pelabresib was formulated as both capsules and tablets for oral administration. The capsule formulation consisted of CPI-0610 monohydrate (2, 10, or 25 mg), microcrystalline cellulose PH101, and magnesium stearate in hydroxypropyl methyl cellulose capsules. A tablet formulation was introduced after the study had been initiated which consisted of micronized CPI-0610 monohydrate (25 mg) and inactive excipients that included microcrystalline cellulose, lactose monohydrate, hydroxypropyl cellulose, croscarmellose sodium, sodium lauryl sulfate, colloidal silicon dioxide, and magnesium stearate.

Pelabresib was administered once daily (QD) for 14 days followed by a 7-day break, in continuous 21-day cycles to prevent/ameliorate thrombocytopenia. Cohorts of 3 to 6 patients were sequentially enrolled with increasing doses of pelabresib (6, 12, 24, 48, 80, 120, 170, 230, and 300 mg with the capsule formulation and 125 mg and 225 mg with the micronized tablet formulation) until the MTD was determined. Pelabresib was administered under fasting conditions (no food intake 2 hours before until 1 hour after study drug administration).

#### Endpoints

The primary objective of the study was to determine the MTD of pelabresib with characterization of dose-limiting toxicities (DLT) as a primary endpoint. Secondary objectives included adverse events (AE) and serious adverse events (SAE); tumor response according to 2007 Revised Response Criteria for Malignant Lymphoma (13); plasma concentration of pelabresib; PK parameters; changes in expression of *MYC* and other genes sensitive to BET inhibition; and changes in levels of selected mRNAs that are expressed in PBMCs and are sensitive to BET inhibition.

To characterize the plasma PK of pelabresib for the first dose and at steady state for multiple dosing, blood samples were taken before and at 0.5, 1, 1.5, 2, 3, 4, 6, 8, and 24 hours after the initial dose; before dosing on Day 8; before and at 0.5, 1, 1.5, 2, 3, 4, 6, 8, 24, 48, 72, 96, and 120 hours after the Day 14 dose; and prior to dosing on Day 1 of Cycle 2. Blood samples were centrifuged within 30 minutes of collection to harvest the plasma which was promptly stored in cryovials maintained at −80°C. The concentration of CPI-0610 in plasma samples was determined by reverse-phase high performance liquid chromatography with tandem mass spectrometric detection. The assay was validated and applied to the analysis of study samples according to the recommendations in the current FDA guidance. At the lowest concentration included in the calibration curves, which was 0.25 ng/mL, interday accuracy was within 0.3% of the nominal concentration and the precision was 3.0%. Interday accuracy ranged from 99.9 to 101.2% and the precision ranged from 4.0 to 6.0% for all other calibration standards. Actual time points were calculated as the difference between the blood sample collection time and the time that the drug was taken. The plasma concentration–time data were analyzed by noncompartmental methods using WinNonlin version 5.0.1 (Pharsight Corp). Area under the plasma concentration–time curve from the time of dosing to the time of last sample collected prior to administration of the next dose (AUC_0–24_) was estimated using the linear-log trapezoidal method. Apparent oral clearance (CL/F) was calculated as the dose divided by the AUC_0–24_ for the dose given on Day 14. Pharmacokinetic parameters are reported as the geometric mean [geometric coefficient of variation (CV%)] of the values for individual patients at each dose level unless otherwise indicated.

Whole (peripheral) blood samples were also collected at selected time points that coincided with PK sampling. Blood from patients was drawn into PAXgene blood RNA tubes (Becton Dickinson) at the clinical sites and shipped frozen to the Massachusetts General Hospital (MGH, Boston, MA) for processing. RNA was purified using a PAXgene Blood RNA Kit IVD (Qiagen) according to the manufacturer's protocol and quantitated using a Nanodrop 8000 (Thermo Scientific). RNA isolated from PBMCs was used to synthesize cDNA for qPCR analysis and gene expression was assessed by qPCR using a *CCR1* or *IL8* TaqMan assay. Housekeeping genes were assessed using *B2M* TaqMan assay, *PPIB* Universal Probe Library (UPL) or TaqMan assay. Relative gene expression for *CCR1* and *IL8* was determined after normalization to the geometric mean of the two housekeeping genes (*B2M* and *PPIB*).

### Statistical Analysis

The MTD of pelabresib was defined as the highest dose that could be given without causing DLT in 33% or more of the patients treated at that dose (capsules or tablets). Dose-escalation followed a traditional “3 + 3” design based on the frequency of DLT. All patients who were treated with at least 1 dose of pelabresib were included in the Safety Population and used for all tabulations of demographic information, baseline characteristics, safety, PK, pharmacodynamic, and efficacy analyses.

### Data Availability Statement

The data generated in this study are not publicly available due to patient privacy but are available upon reasonable request from the corresponding author.

### Study Conduct

The study was approved by the institutional review board at each participating site and was conducted in accordance with the principles of the Declaration of Helsinki. The study was overseen by an independent safety review committee. Written informed consent was obtained from each patient or from the patient's legally authorized representative prior to study entry.

Data were collected by the study investigators and analyzed by the study sponsor, Constellation Pharmaceuticals. All of the authors, including authors employed by the study sponsor, contributed to authoring the manuscript for publication.

## Results

### Patient Disposition and Demographics

Between September 2013 and December 2017, 64 patients were enrolled and treated with pelabresib. Forty-seven patients enrolled in the study were treated with the capsule formulation of pelabresib at 9 dose levels ranging from 6 mg to 300 mg QD (Supplementary Fig. S1). Seventeen patients received the tablet formulation at 125 mg (*n* = 7) and 225 mg (*n* = 10) daily. As 1 of the initial 3 patients experienced a DLT, additional patients were dosed in the 6, 48, 80, 170, and 230 mg capsule and 125 mg tablet cohorts. None of these additional patients experienced DLTs and dose escalation was able to continue until the MTD definition was met.

All patients discontinued pelabresib treatment and progressive disease was the most commonly reported reason for treatment discontinuation (50 patients, 78.1%). The remaining 14 pts discontinued pelabresib treatment either due to AE, PI decision, patient consent withdrawal or death.

In total, 5 (7.8%) patients discontinued from the study, 1 each due to no active lymphoma, alternative treatment, death from progressive disease, AEs or laboratory abnormalities (grade 3 AEs of hyponatremia, confusional state, and hypertension) and disease progression, respectively.

The median age was 63 years, and the most common lymphoma subtype was diffuse large B-cell lymphoma (56.3%), followed by follicular lymphoma (12.5%), and Hodgkin lymphoma (7.8%). Patients had received a median of 4 prior therapies, and the mean time from diagnosis to study entry was 42.62 months (Table 1).

**TABLE 1:** Demographic characteristics and disease characteristics

Characteristic	Overall *N* = 64
Sex, *n* (%)	
Male	48 (75.0)
Female	16 (25.0)
Race, *n* (%)	
White/Caucasian	60 (93.8)
Asian	2 (3.1)
Other	2 (3.1)
Ethnicity, *n* (%)	
Latino/Hispanic	4 (6.3)
Not Latino/Hispanic	59 (92.2)
Declined	1 (1.6)
Age (years)	
Median	63.0
Min, Max	23, 92
Lymphoma type, *n* (%)	
Diffuse large B-cell lymphoma	36 (56.3)
Follicular lymphoma	8 (12.5)
Hodgkin lymphoma	5 (7.8)
Mantle cell lymphoma	3 (4.7)
Other	3 (4.7)
Burkitt lymphoma	2 (3.1)
Mycosis Fungoides	2 (3.1)
Nodal marginal zone B-cell lymphoma	2 (3.1)
Lymphoplasmacytic lymphoma	1 (1.6)
Peripheral T-cell lymphoma (not otherwise specified)	1 (1.6)
Small lymphocytic lymphoma	1 (1.6)
Time since initial diagnosis (months)	
Mean (STD)	42.62 (42.416)
Median	29.45
Min, Max	4.4, 240.8
Number of lines of prior systemic therapy (for lymphoma)	
Median	4.0
Min, Max	1, 12
1	2 (3.1)
2	14 (21.9)
3	14 (21.9)
≥4	34 (53.1)

Abbreviations: Max, maximum; Min, minimum; STD, standard deviation.

### Safety

All 64 patients in the safety analysis set received at least one dose of pelabresib. Thirty-seven of 64 (58%) patients completed 1 cycle of treatment and 42% completed 2 cycles. Median number of cycles completed was 2 (range: 1 to 66). The proportion of patients treated with 2 cycles was similar between capsule and tablet formulations (Supplementary Table S1). The median relative dose intensity was generally high (approximately 70% to 100%) and consistent across treatment cycles 1 to 3.

All 64 patients had at least 1 treatment-emergent adverse event (TEAE) during the study; 44 (68.8%) patients had at least 1 TEAE of Grade 3 or higher intensity. Treatment-emergent AEs that led to treatment discontinuation were reported for 9 (14.1%) patients: 3 from progressive disease; 2 from elevated liver enzymes; 1 from hyponatremia, confusion, and hypertension and 1 each from diarrhea, fatigue, and thrombocytopenia. Only disease progression led to treatment discontinuation in more than 2 patients (3 [4.7%] patients). In total, 9 (14.1%) patients died while on study, for all of whom the cause of death was reported as disease progression; none of these deaths were considered related to the investigational product.

The most frequently reported TEAEs (≥15%) regardless of relationship to study drug are presented in Table 2. Hematologic TEAEs occurring in more than 2 patients in any dose group were anemia, thrombocytopenia events, and lymphopenia. Hematologic TEAEs with an intensity of Grade 3 or higher included thrombocytopenia events in 13 (20.3%) and anemia in 7 (10.9%) patients. Thrombocytopenia led to discontinuation of pelabresib for 1 patient in the 125 mg tablet dose group.

**TABLE 2:** Summary of most frequently reported treatment-emergent adverse events regardless of relationship to study drug

	Capsule formulation									Tablet formulation		
System organ class preferred term	6 mg *N* = 5 *n* (%)	12 mg *N* = 3 *n* (%)	24 mg *N* = 3 *n* (%)	48 mg *N* = 7 *n* (%)	80 mg *N* = 6 *n* (%)	120 mg *N* = 4 *n* (%)	170 mg *N* = 7 *n* (%)	230 mg *N* = 9 *n* (%)	300 mg *N* = 3 *n* (%)	125 mg *N* = 7 *n* (%)	225 mg *N* = 10 *n* (%)	Overall *N* = 64 *n* (%)
**Hematologic events**												
Thrombocytopenia events^a^	0	0	1 (33.3)	1 (14.3)	3 (50.0)	2 (05.0)	4 (57.1)	6 (66.7)	2 (66.7)	4 (57.1)	6 (60.0)	29 (45.3)
Anemia	1 (20.0)	1 (33.3)	0	2 (28.6)	1 (16.7)	2 (50.0)	0	4 (44.4)	1 (33.3)	2 (28.6)	2 (20.0)	16 (25.0)
Lymphopenia	0	0	0	1 (14.3)	2 (33.3)	1 (25.0)	1 (14.3)	4 (44.4)	0	1 (14.3)	3 (30.0)	13 (20.3)
**Nonhematologic events**												
Gastrointestinal disorders												
Nausea	1 (20.0)	2 (66.7)	1 (33.3)	0	1 (16.7)	0	0	3 (33.3)	2 (66.7)	4 (57.1)	4 (40.0)	18 (28.1)
Vomiting	1 (20.0)	1 (33.3)	1 (33.3)	1 (14.3)	3 (50.0)	1 (25.0)	0	2 (22.2)	0	0	3 (30.0)	13 (20.3)
Diarrhea	1 (20.0)	0	0	0	0	1 (25.0)	3 (42.9)	2 (22.2)	2 (66.7)	1 (14.3)	2 (20.0)	12 (18.8)
Other nonhematologic events												
Fatigue	2 (40.0)	1 (33.3)	1 (33.3)	3 (42.9)	2 (33.3)	2 (50.0)	1 (14.3)	3 (33.3)	0	3 (42.9)	5 (50.0)	23 (35.9)
Decreased appetite	0	0	0	2 (28.6)	3 (50.0)	1 (25.0)	1 (14.3)	2 (22.2)	0	3 (42.9)	5 (50.0)	17 (26.6)
Hyperglycemia	1 (20.0)	1 (33.3)	0	0	0	1 (25.0)	1 (14.3)	2 (22.2)	1 (33.3)	1 (14.3)	4 (40.0)	12 (18.8)
Dysgeusia	0	0	0	0	1 (16.7)	0	0	3 (33.3)	1 (33.3)	1 (14.3)	4 (40.0)	10 (15.6)

^a^Include MedDRA PTs platelet count decreased and thrombocytopenia.

Gastrointestinal TEAEs occurred in 43 (67.2%) patients, with at least 1 TEAE occurring in each dose group. Gastrointestinal TEAEs occurring in more than 2 patients in any dose group were nausea, vomiting, and diarrhea. Of these, diarrhea was the only TEAE with an intensity of Grade 3 or higher, which occurred in 4 (6.3%) patients (all were treated with the capsule formulation of pelabresib). Diarrhea also led to discontinuation of pelabresib for 1 patient in the 230 mg capsule dose group. Other non-hematologic TEAEs occurring in more than 2 patients in any dose group were fatigue, decreased appetite, hyperglycemia, and dysgeusia.

Treatment-emergent SAEs were reported for 27 (42.2%) patients. Serious AEs reported for more than 1 patient (in descending order) were disease progression (7 patients grade 5 and 1 patient grade 3), diarrhea (4 patients grade 3), acute renal failure (1 patient grade 4 and 2 patients grade 2), pain (2 patients grade 3), and pneumonia (2 patients grade 3).

There was no apparent dose relationship for most TEAEs; however, a pattern is difficult to detect owing to the small number of patients in each dose group.

**FIGURE 1:**
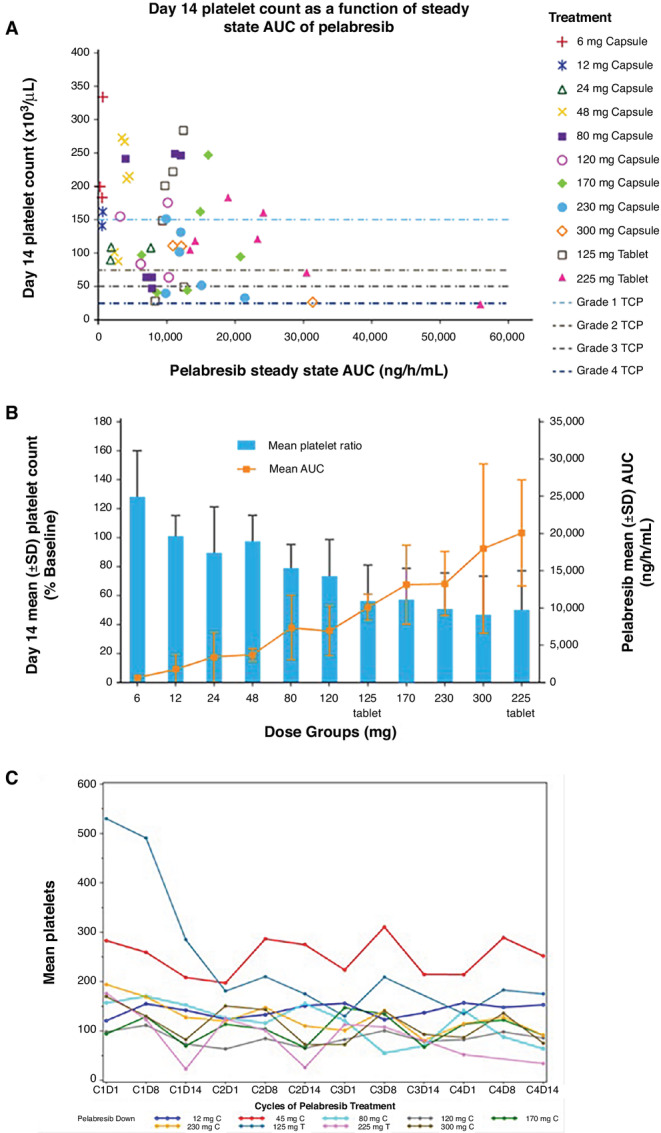
Relationship between pelabresib and platelet count values. **A,** Day 14 platelet count as a function of steady state AUC of pelabresib. **B,** Day 14 mean platelet count as a percent of baseline compared with pelabresib mean AUC. The coefficient of determination (*R*^2^) = 0.8122, obtained from a simple linear regression model with mean platelet count ratio as the dependent variable and pelabresib capsule dose as the independent variable (tablet doses were multiplied by 1.34 to convert to capsule doses). **C,** Mean platelet count over time. AUC, area under the curve; CPI-0610, pelabresib; CXDX, Cycle number Day number; X mg C, Capsule pelabresib dose; X mg T, Tablet pelabresib dose.

The relationship between platelet count values on Cycle 1 Day 1 and Cycle 1 Day 14 and pelabresib treatment group is displayed in Fig. 1A and 1B. To accommodate for different baseline platelet count values in different patients, changes in platelet count values were expressed as a fraction or ratio of the baseline value. There was a strong correlation between the Day 14:Day 1 platelet count ratio and pelabresib dose (*R*^2^ = 0.81).

When examining platelet count values from individual patients over the course of several cycles, it appeared that after the decline, platelet count values rebounded within the next cycle of treatment, typically peaking by the end of the first week of the next cycle (i.e., Cycle 2, Day 8; Fig. 1C). This trend continued throughout multiple cycles of pelabresib treatment.

### Dose Limiting Toxicities

Fifty-six out of the 64 treated patients were evaluable for DLT determination (Supplementary Table S2). No DLTs were observed in the 15 evaluable patients treated at 6 mg, 12 mg, 24 mg and 120 mg capsule cohorts. One DLT each was observed in the first 3 evaluable patients treated at 48 mg, 80 mg, 170 mg, 230 mg and 300 mg capsule cohorts, and at 125 mg and 225 mg tablet cohorts. These cohorts were then expanded to additional patients except the 300 mg capsule cohort as a new micronized tablet formulation was introduced at that time.

One out of 7 evaluable patients treated at 225 mg QD tablet cohort developed a DLT (Grade 4 platelet count decrease; Supplementary Table S2), which ordinarily would have led to escalation to the next, higher dose cohort; however, based on the PK/PD, safety, and clinical activity results combined with the results of the other concurrently conducted phase 1 study 0610–02 in patients with myelodysplastic syndromes/myeloproliferative neoplasms (MDS/MPN), the 225 mg QD tablet dose was determined to be the MTD.

A total of 8 patients had 8 DLTs during the DLT assessment period (Cycle 1; Supplementary Table S2). These 8 DLTs included hematologic events (*n* = 5), gastrointestinal (GI) events (*n* = 2), and rash (*n* = 1).

Three of five hematologic DLT events were Grade 4 thrombocytopenia events (include MedDRA PTs of platelet count decreased and thrombocytopenia). These DLTs of thrombocytopenia events were dose dependent in both frequency and intensity, reversible, noncumulative and not associated with bleeding events. Of these 3 thrombocytopenia events, one event did not require a dose reduction; one required a dose reduction (from 300 mg QD capsule to 170 mg QD capsule); and the other required a dose interruption for approximately 2 weeks, followed by a dose reduction in the following cycle (from 225 mg to 150 mg QD tablet). The remaining two hematologic DLT events were neutropenia, reported for 2 patients: Grade 3 febrile neutropenia lasting for 4 days in 1 patient (dose unchanged from 80 mg QD capsule) and 1 Grade 4 neutropenia lasting for 3 days in the second patient (dose interrupted for approximately 1 week, followed by the same dose of 125 mg QD tablet in the following cycle); Grade 4 neutropenia was not associated with fever, infections or any serious consequences and resolved in 2 days with dose interruption; both neutropenia DLT events were resolved.

The 2 gastrointestinal DLT events were grade 3 diarrhea reported for 2 patients. One patient had diarrhea for 2 days associated with dehydration, required withdrawal of pelabresib (230 mg QD capsule), and the second patient had diarrhea for 7 days associated with dehydration requiring i.v. fluids, did not require a change in dosing (170 mg QD capsule). Both gastrointestinal DLT events were resolved.

The remaining DLT event was a grade 3 erythematous maculopapular rash covering approximately 40% of total body surface area for 11 days that required systemic steroids and a dose interruption for approximately 1.5 weeks, followed by a dose reduction in the following cycle (from 48 mg QD to 24 mg QD capsule).

### Antitumor Activity

All 64 patients included in the Safety Population were assessed for tumor response.

The overall response rate [complete response (CR) + partial response (PR)] was 6.2% (4 of 64 patients; Table 3) in this heavily pretreated population (95.3% of overall patients had at least 1 prior chemotherapy). The best overall response (BOR) included CR observed in 2 (3.1%) patients (1 with T-cell/histocyte-rich DLBCL treated with 48 mg capsule (Supplementary Fig. S2), 1 with ABC-DLBCL treated with 230 mg capsule) and PR observed in 2 (3.1%) patients (1 with follicular lymphoma treated with 230 mg capsule, 1 with ABC-DLBCL treated with 300 mg capsule). It is worth noting that all patients that achieved CR or PR had previously been treated with at least 2 prior chemotherapy regimens. A total of 21 (32.8%) patients achieved stable disease as their BOR; 5 patients (3 patients with follicular lymphoma and 1 patient each with nodal marginal zone B-cell lymphoma and diffuse large B-cell lymphoma) had prolonged (>6 months) stable disease.

**TABLE 3:** Demographic characteristics and disease characteristics

Response	Overall *N* = 64
Overall response rate (CR + PR), *n* (%)	4 (6.2)
Best overall response, *n* (%)	
CR	2 (3.1)
PR	2 (3.1)
SD	21 (32.8)
Time to response (CR or PR), cycles	2–6
Median duration of response (CR or PR), weeks (range)	8.4 (6.1–46.1)
Time to treatment (CR or PR), cycles	4–18

NOTE: Tumor response was determined according to the 2007 Revised Response Criteria for Malignant Lymphoma.

Abbreviations: CR, complete response; PR, partial response; SD, stable disease.

Patients who achieved CR or PR had a time to response between 2 and 6 cycles [CR: 4 (77 days) and 6 (129 days) cycles; PR: 2 (57 days) and 4 (80 days) cycles], median duration of response was 8.4 weeks (range: 6.1 weeks to 46.1 weeks), and remained on treatment for a range of 4 to 18 cycles.

### Pharmacokinetics

**FIGURE 2:**
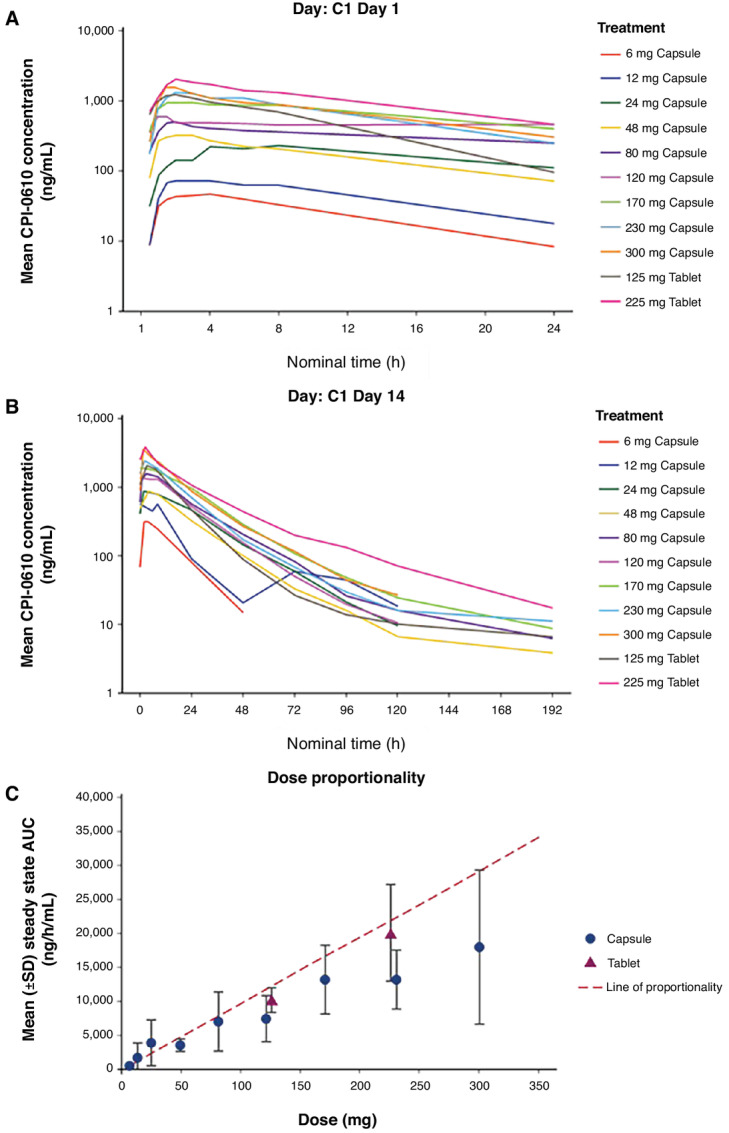
Mean pelabresib plasma profiles and dose proportionality. **A,** Mean pelabresib plasma profile over time at Cycle 1 Day 1. **B,** Mean pelabresib plasma profile over time at Cycle 1 Day 14. **C,** Dose proportionality of mean steady-state AUC of patients treated with capsule and tablet doses of pelabresib. Subjects receiving a dose other than the planned dose were excluded from the calculation of concentration summary statistics; Predose/negative nominal time were set to 0.

Mean plasma concentration–time profiles of pelabresib for the groups of patients evaluated at each dose level are shown in Fig. 2A for the initial dose and in Fig. 2B for the Day 14 dose. Pelabresib was rapidly absorbed with peak plasma concentrations occurring at a median time of 1.9 hours for both the initial dose (range: 0.5– 7.4 hours) and the Day 14 dose (range: 0.9–7.9 hours). Plasma concentrations declined from the peak in a manner that appeared to be biphasic for the majority of patients. Across the capsule dose range studied, pelabresib exposure increased with dose in an approximately proportional manner up to 170 mg (Fig. 2C) and exhibited a trend toward saturation of absorption at capsule doses greater than 170 mg. The micronized tablet formulation, developed to address potential solubility limitations of the capsule formulation and minimize the number of capsules, was tested after the 300 mg capsule cohort. Pelabresib exposure was approximately 60% greater for the tablet as compared with the capsule (Fig. 2C) and was selected for use in subsequent clinical trials. The geometric mean (geometric CV) terminal half-life of pelabresib was 11.6 (48.2%) hours for the capsule and 15.3 (45.0%) hours for the tablet. Following 14 days of continuous QD dosing, the mean (±SD) accumulation of pelabresib was minimal, 20.5 ± 7.2% for the capsule formulation and 4.3 ± 1.0% for the tablet, consistent with the estimated half-life of each formulation and confirming the suitability of a once-daily dosing schedule. The geometric mean apparent oral clearance of pelabresib in patients receiving the tablet dosage form was 12.3 (27.2%) L/h. In the 10 patients with lymphoma who were treated with the MTD dose of 225 mg QD, the mean maximum plasma concentration (C_max_) and AUC_0–24_ after the first dose was 2,021 ng/mL and 20,700 ng/h/mL, respectively, which were higher than the mean respective values achieved with capsule doses of 170, 230, and 300 mg QD.

### Pharmacodynamics

Based on a whole blood assay developed in preclinical studies, *IL8* and *CCR1* were the most strongly BET-regulated genes exhibiting the strongest exposure dependent downregulation. Thus, examination of these genes in patient samples allowed for the identification of the minimum threshold of exposure required for BET target engagement.

**FIGURE 3:**
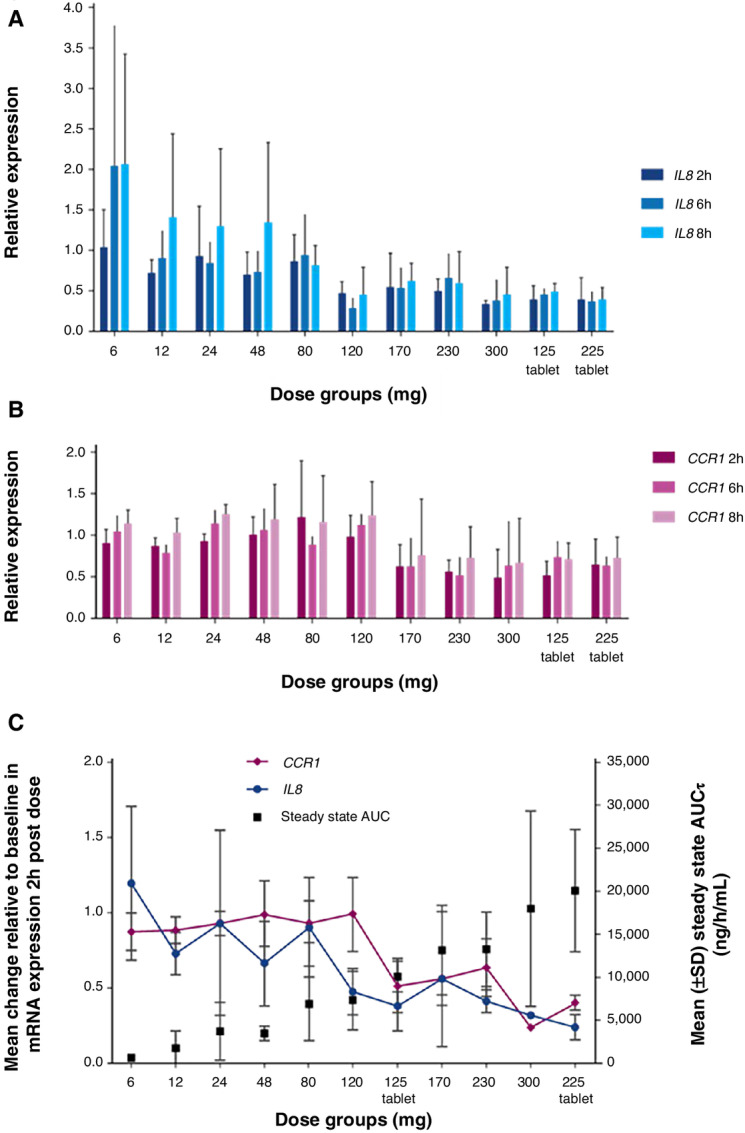
Gene expression analysis of *IL8* and *CCR1* transcript levels after treatment with pelabresib. **A,** Relative expression of *IL8* blood mRNA levels prior to and 2, 6, and 8 hours after pelabresib treatment. **B,** Relative expression of *CCR1* blood mRNA levels prior to and 2, 6, and 8 hours after pelabresib treatment. Relative mRNA expression of *IL8* and *CCR1* after dosing is shown relative to the pretreatment values (y axis). **C,** Relative *IL8* and *CCR1* mRNA expression compared to the Cycle 1 Day 14 steady-state AUC. *IL8* and *CCR1* are represented as the average at the 2-hour time point following pelabresib administration over that of baseline in each dose group. The steady state (C1D14) AUC0–24 (hour/ng/mL, geometric mean of each dose level) is shown on the right *y* axis. T = tablet 125 mg and 225 mg were tablet formulations; all other doses were capsule formulation.

The expression of *IL8* (Fig. 3A) and *CCR1* (Fig. 3B) in patient whole blood at 2 hours, 6 hours and 8 hours after administration of pelabresib was tested to understand the PD effect of pelabresib. Patients from dose cohorts above the 120 mg capsule dose showed appreciable changes in the expression of *IL8*, while patients from dose cohorts above the 170 mg capsule dose showed appreciable changes in expression of *CCR1* after administration of pelabresib. Patients treated with both tablet doses showed appreciable changes in the expression of *IL8* and *CCR1* after administration of pelabresib. Although there is some variation in expression at lower doses, suppression of *IL8* and *CCR1* was much more apparent at exposures conferred by capsule doses greater than or equal to 120 and 170 mg doses, respectively, including both tablet dose levels of pelabresib. The maximum reduction of *IL8* expression was observed in patients treated at the 120 mg capsule dose level which approximately corresponds to a 75 mg tablet dose. Decreased expression of both *IL8* and *CCR1* are seen as early as 2 hours postdose, suggesting rapid transcriptional regulation following treatment. The extent of the decrease of *IL8* and *CCR1* gene expression observed in patients achieving a CR or PR was consistent with the effect observed in other nonresponding patients treated at the same dose levels.

Given that pelabresib exposure increased as dose increased, the relationships between gene expression of *IL8* and *CCR1* and both pelabresib dose and exposure (AUC_0–24_) were evaluated. *IL8* and *CCR1* expression change at the 2-hour timepoint postdose was selected as the inter-patient variation of both genes was less than the 6- or 8-hour timepoints. As shown in Fig. 3C, there is a correlation between suppression of *IL8* or *CCR1* and exposure to pelabresib, as represented by the geometric mean of pelabresib AUC_0–24_ at each dose level. Suppression of BET target gene expression increased with dose escalation.

To further explore the relationship between pelabresib exposure and suppression of gene expression, individual maximum gene suppression (the nadir of individual gene expression versus time profiles) was plotted as a continuous function of individual C_max_ and AUC for both *CCR1* and *IL8*. An inhibitory maximum effect (E_max_) model of drug effect was fit to the data and demonstrates that both *IL8* and *CCR1* expression are clearly reduced as pelabresib exposure increases (Supplementary Fig. S3). Examination of the curves and the half-maximal inhibitory concentration (IC_50_) values shows that *IL8* gene expression is more sensitive (steeper relationship and left-shifted) compared with *CCR1* gene expression in the pelabresib concentration range studied. Of note, while the model fit for *CCR1* exhibited high coefficient of variation on E_max_ and IC_50_, indicating the data may be insufficient to fully represent the dynamic range for this gene, the trend of suppression with increasing exposure is clear even if not precise. In total, based on this analysis, pharmacodynamic responses of at least 50% suppression in gene expression are evident at AUC and C_max_ as low as 4,000 ng/hr/mL and 500 ng/mL, respectively. These exposures were achieved on average at capsule doses of 80 mg and above and were exceeded at both tablet doses.

## Discussion

On the basis of these results, pelabresib (previously known as CPI-0610) has potential antitumor activity, particularly in heavily pretreated patients with *NF-kB*-driven relapse/refractory lymphoma, at doses below the MTD with clear BET-driven target suppression and appeared to have an acceptable safety profile. All of the doses of pelabresib demonstrated rapid absorption and a half-life that supported daily dosing. Suppression of BET target genes was much more apparent at exposures conferred by capsule doses greater than or equal to 170 mg including both tablet dose levels of pelabresib. Decreases in platelet count values were dose dependent, reversible, and non-cumulative. Based on the PK/PD, safety, and clinical activity results together with the other concurrently conducted phase 1 study 0610–02 in patients with MDS/MPN, the 225 mg QD tablet dose was determined to be the MTD. Taking into account the evidence for pelabresib to suppress gene expression and platelet count values, the starting dose selected for phase 2 studies (RP2D) was the 125 mg QD tablet dose for 14 days with a 7-day break. This dose regimen provided PK parameters leading to sustained BET target gene suppression with a manageable safety profile; including changes in platelet count values.

The starting dose for this study, 6 mg orally QD, took into account the results of toxicology studies in dogs and rats as well as predictions of the PK of pelabresib in humans. Although standard starting dose calculations suggested a dose of 23 mg/day (1/6 of the highest non-severely toxic dose in the dog), predictions of human PK suggested that a dose as low as 15 mg/day could achieve therapeutic exposures to pelabresib. Since therapeutic and toxic exposures were expected to be similar, the starting dose in this study was therefore lowered to 6 mg/day. For the capsule formulation of pelabresib, 230 mg was considered the MTD (2 patients experienced a DLT out of 9). This data suggested that the MTD was much higher than the dose estimated to achieve therapeutic exposures to pelabresib.

Initially, patients were enrolled to the study and treated with the capsule formulation of pelabresib, but the new micronized tablet formulation, designed to address potential solubility limitations of the capsule formulation and minimize the number of capsules, was introduced toward the end of enrollment to the 300 mg capsule cohort. The PK profile of the tablet formulation was similar to that of the capsule. Maximum plasma concentrations were reached at a median time of 2 hours and the mean apparent terminal phase half-life was 15 hours, supporting QD dosing. Dose proportional increases in systemic exposure were observed within the dose range of 6 to 170 mg QD administered in capsule form; however, AUC values were similar across the 170 to 300 mg dose range, suggesting a limitation on drug absorption, potentially related to the low aqueous solubility of the crystalline form of pelabresib. The tablet formulation with micronized drug substance had improved oral exposure relative to the higher capsule doses of pelabresib.

Dose-related thrombocytopenia, a class effect for all BET inhibitors (14), was the principal DLT. In this study, the greatest decrease of platelet counts generally occurred during the first treatment cycle. Decreases in platelet count values were reversible as platelet count values tended to decrease during dosing days and to stabilize or recover during periods of dosing interruption. Changes in platelet count values were also dose dependent as there was a strong correlation between change in platelet count from baseline to the end of the first treatment cycle and pelabresib treatment group. Taken together, thrombocytopenia was dose dependent, in both frequency and intensity, and was reversible and non-cumulative, and these data supported the dosing schedule of pelabresib QD for 14 days, followed by a 7-day break, with cycles of treatment repeated every 21 days. The 7-day break from treatment built into each cycle allowed for recovery from on-target normal tissue toxicity yet maintain pharmacologically active doses of pelabresib.

The dosing regimen of pelabresib QD for 14 days, followed by a 7-day break was supported by the data in this study. This dosing regimen was initially chosen based on the aim of achieving continuous inhibition of the expression of *MYC* and other genes (like B-cell lymphoma 2) for approximately 2 weeks, since preclinical studies suggested longer exposure times are associated with greater antitumor activity. The 7-day break permitted recovery from on-target normal tissue toxicity, including thrombocytopenia observed in preclinical toxicology studies. In the current study, accumulation of pelabresib was minimal (approximately 40% at most) and with its estimated half-life confirmed the suitability of a once-daily for 14 days dosing schedule. The reversibility of decreased platelet count values with the 7-day break further supported the dosing regimen of pelabresib QD for 14 days, followed by a 7-day break.

In progressive lymphoma patients, the QD and efficacy data suggest doses well below the MTD could provide therapeutic benefit as a single agent. Suppression of *IL8* and *CCR1* was apparent at exposures conferred by capsule doses greater than or equal to 120 and 170 mg doses of pelabresib (including both tablet doses), respectively. It was observed that as exposure increased with dose escalation, suppression of BET target gene expression was increased as well. Patients treated with as low as 48 mg QD pelabresib achieved complete remission during the study. This preliminary efficacy data suggests modest clinical activity at doses lower than the MTD with acceptable PK and PD parameters and less hematological toxicity; thus, it is conceivable lower dose pelabresib may confer antitumor activity with a reasonable adverse event profile when paired with other agents.

This was a phase 1 study designed to evaluate safety and MTD, which also shed light on pelabresib exposure and pharmacodynamic effects in humans. Further exploration of the efficacy of this treatment is warranted. Because thrombocytopenia is a class effect known for BET inhibitors and decreases in platelet count values were dose dependent and BET target gene suppression was observed with both tablet doses, the lower 125 mg tablet dose was chosen for future studies.

There are currently no ongoing studies in lymphomas, but the 125 mg tablet dose of pelabresib is currently being studied as monotherapy and in combination with ruxolitinib in phase 2 (MANIFEST NCT02158858) and phase 3 (MANIFEST-2 NCT04603495) studies of individuals with myelofibrosis. Pelabresib regulates megakaryocyte differentiation and proliferation and reduces proinflammatory cytokine levels resulting in a potential change of the natural course of clonal myelofibrosis (15, 16). Kleppe and colleagues have shown that these benefits increased when BET inhibition was combined with ruxolitinib, suggesting synergism by cooperated downregulation of JAK-driven oncogenic activity and BET-driven proinflammatory signaling (17).

In this first-in-human dose escalation study in patients with lymphomas, including non-Hodgkin or Hodgkin lymphoma, all doses of pelabresib demonstrated rapid absorption and a half-life that supported daily dosing. Pelabresib demonstrated antitumor activity with an overall response rate of 6.2% in these heavily pretreated patients with refractory/relapsed lymphomas. The 225 mg pelabresib tablet QD was the MTD and doses above the 170 mg capsule (including both the 125 and 225 mg tablet doses QD) were capable of suppressing BET target genes and appeared to have an acceptable safety profile. Thrombocytopenia was dose dependent, non-cumulative, reversible, and manageable with QD treatment for 14 days with a 7-day break. Both doses of the tablet formulation are capable of BET target gene suppression but due to the dose dependent nature of the observed platelet count decrease the 125 mg tablet dose has a better safety profile. Thus, the RP2D of pelabresib in myelofibrosis development is 125 mg tablet QD for 14 days with a 7-day break and ongoing phase 3 registrational studies in myelofibrosis in combination with ruxolitinib is being tested.

## Supplementary Material

Supplementary Data Supplemental tables with the number of patients treated with number of cycles and the incidence of DLTs.

Figure S1 CONSORT diagram of patient disposition throughout study

Figure S2 Representative image of complete response after treatment with pelabresib.

Figure S3 PK/PD Analysis of the Relationship Between Maximum Relative Change in Gene Expression and Pelabresib Exposure Metrics.
