# Supplementary material for: A Phase I Study of Pelabresib (CPI-0610), a Small-Molecule Inhibitor of BET Proteins, in Patients with Relapsed or Refractory Lymphoma
Source: Cancer Res Commun. 2022 Aug 11;2(8):795–805. doi: 10.1158/2767-9764.CRC-22-0060 (PMC10010313; doi:10.1158/2767-9764.CRC-22-0060)
Supplement: Supplementary Data — Supplemental tables with the number of patients treated with number of cycles and the incidence of DLTs. [file crc-22-0060-s01.docx]

**Supplemental Materials**

**Supplemental Table 1 Patients Treated with Number of Cycles of Pelabresib by Formulation**

| Cycle | Formulation | |
| --- | --- | --- |
|  | Capsule | Tablet |
| 1 | 47 (100.0) | 17 (100.0) |
| 2 | 20 (42.6) | 7 (41.1) |
| 3 | 15 (31.9) | 2 (11.8) |
| >4 | 12 (25.5) | 0 (0.0) |

**Supplemental Table 2 Incidence of Dose-Limiting Toxicities**

| **Formulation** | **Dose Cohort (mg)** | **Total subjects treated** | **DLT-evaluable^a^** | **DLTs** | **MedDRA Preferred Term** | **Severity (CTCAE Grade)** | **Action Taken** | **AE Outcome** |
| --- | --- | --- | --- | --- | --- | --- | --- | --- |
| Capsule | 6 | 5 | 5 | 0 |  |  |  |  |
|  | 12 | 3 | 3 | 0 |  |  |  |  |
|  | 24 | 3 | 3 | 0 |  |  |  |  |
|  | 48 | 7 | 6 | 1 | Rash | 3 | Dose interrupted | Resolved |
|  | 80 | 6 | 6 | 1 | Febrile neutropenia | 3 | Dose interrupted | Resolved |
|  | 120 | 4 | 4 | 0 |  |  |  |  |
|  | 170 | 7 | 6 | 1 | Diarrhea | 3 | Dose not changed | Resolved with sequelae |
|  | 230 | 9 | 7 | 2 | Diarrhea | 3 | Drug withdrawn | Resolved |
|  |  |  |  |  | Platelet count decreased | 4 | Drug withdrawn |  |
|  | 300 | 3 | 3 | 1^b^ | Platelet count decreased | 4 | Dose reduced | Resolved |
| Tablet | 125 | 7 | 7 | 1 | Neutropenia | 4 | Dose interrupted | Resolved |
|  | 225 | 10 | 7 | 1 | Platelet count decreased | 4 | Dose interrupted | Resolved |
| Total DLT events considered for dose-escalation | | | | 8 |  |  |  |  |

AE=adverse event; AUC=area under the curve; CTCAE=Common Terminology Criteria for Adverse Events; DLT=dose-limiting toxicity; MTD=maximum tolerated dose.

^a^ DLT-evaluable patients are patients who met the minimum treatment and safety evaluation requirements of the study and/or who experience a DLT during Cycle 1.

^b^ Since a new micronized tablet formulation designed to improve oral bioavailability was introduced at the time the third subject was enrolled in this cohort, and since a capsule formulation of pelabresib will not be further developed, a tablet dose projected to achieve the same average steady-state AUC was evaluated next in a standard cohort of 3-6 subjects. Therefore, the MTD for capsule formulation was not defined.
