# Supplementary material for: A Phase I Study of Pelabresib (CPI-0610), a Small-Molecule Inhibitor of BET Proteins, in Patients with Relapsed or Refractory Lymphoma
Source: Cancer Res Commun. 2022 Aug 11;2(8):795–805. doi: 10.1158/2767-9764.CRC-22-0060 (PMC10010313; doi:10.1158/2767-9764.CRC-22-0060)
Supplement: Figure S1 — CONSORT diagram of patient disposition throughout study [file crc-22-0060-s02.pptx]

## Slide 1
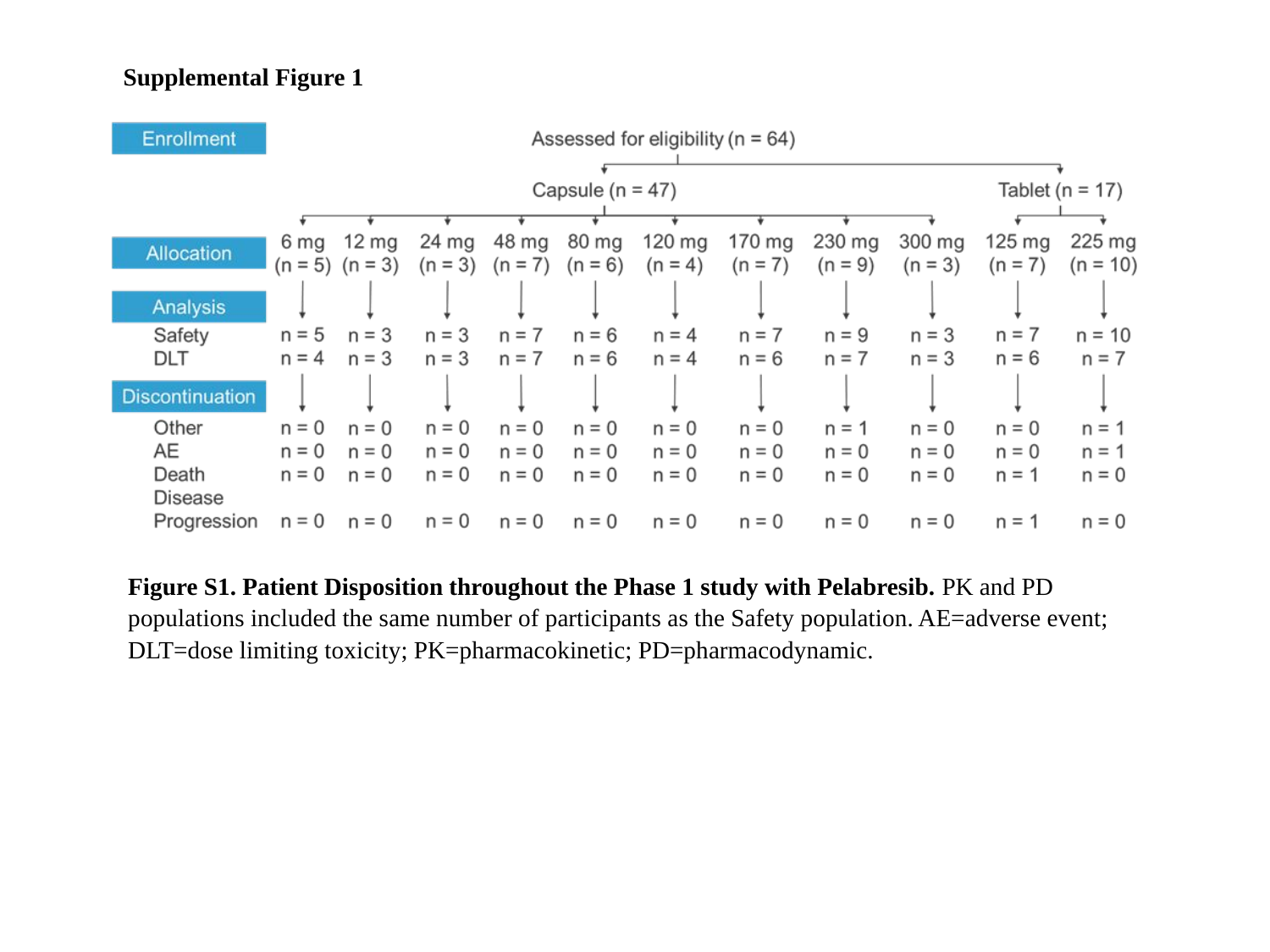

Supplemental Figure 1
Figure S1. Patient Disposition throughout the Phase 1 study with Pelabresib. PK and PD populations included the same number of participants as the Safety population. AE=adverse event; DLT=dose limiting toxicity; PK=pharmacokinetic; PD=pharmacodynamic.
