# Supplementary material for: A Phase I Study of Pelabresib (CPI-0610), a Small-Molecule Inhibitor of BET Proteins, in Patients with Relapsed or Refractory Lymphoma
Source: Cancer Res Commun. 2022 Aug 11;2(8):795–805. doi: 10.1158/2767-9764.CRC-22-0060 (PMC10010313; doi:10.1158/2767-9764.CRC-22-0060)
Supplement: Figure S2 — Representative image of complete response after treatment with pelabresib. [file crc-22-0060-s03.pptx]

## Slide 1
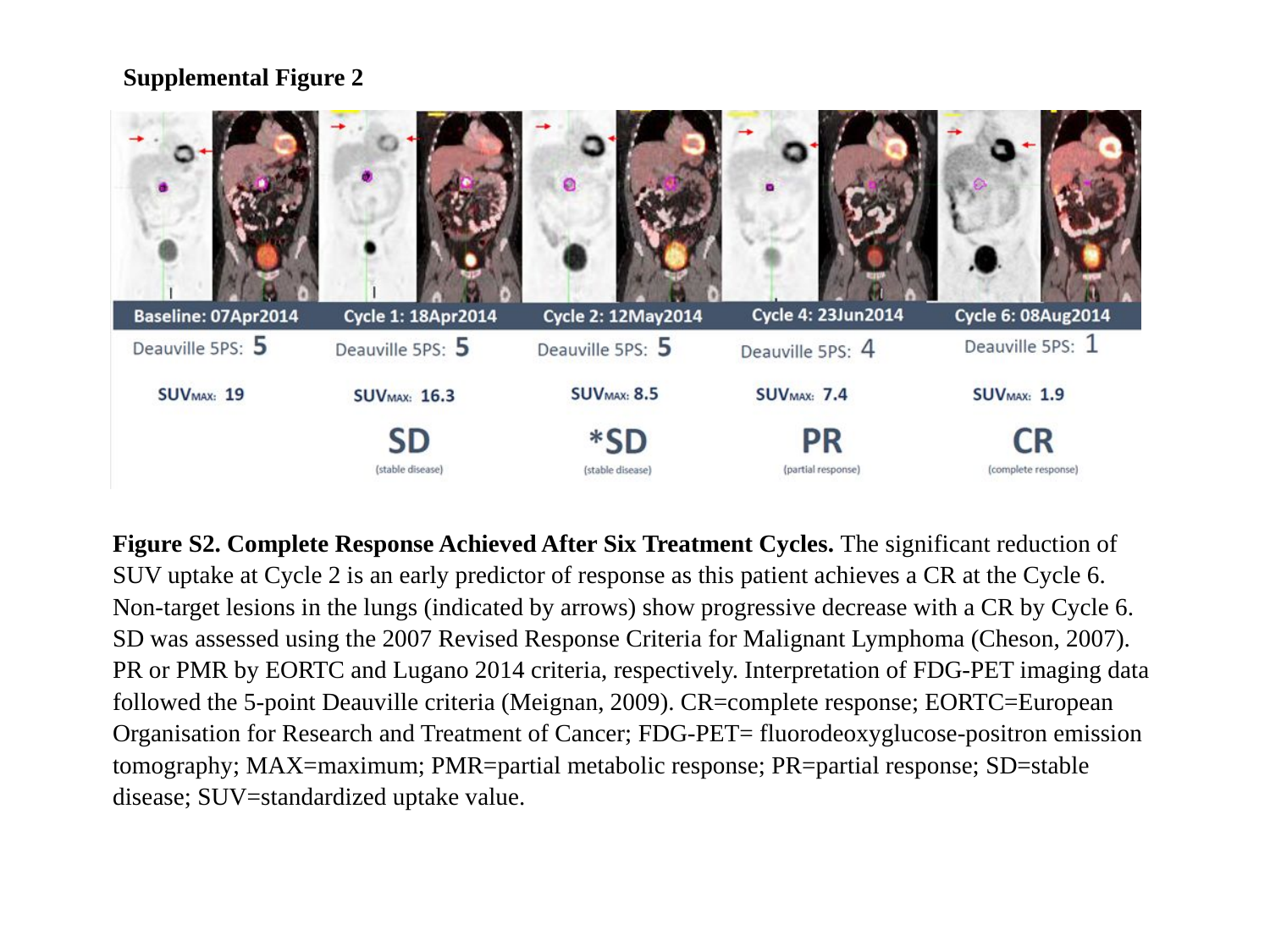

Supplemental Figure 2
Figure S2. Complete Response Achieved After Six Treatment Cycles. The significant reduction of SUV uptake at Cycle 2 is an early predictor of response as this patient achieves a CR at the Cycle 6. Non-target lesions in the lungs (indicated by arrows) show progressive decrease with a CR by Cycle 6. SD was assessed using the 2007 Revised Response Criteria for Malignant Lymphoma (Cheson, 2007). PR or PMR by EORTC and Lugano 2014 criteria, respectively. Interpretation of FDG-PET imaging data followed the 5-point Deauville criteria (Meignan, 2009). CR=complete response; EORTC=European Organisation for Research and Treatment of Cancer; FDG-PET= fluorodeoxyglucose-positron emission tomography; MAX=maximum; PMR=partial metabolic response; PR=partial response; SD=stable disease; SUV=standardized uptake value.
