# Supplementary material for: A Phase I Study of Pelabresib (CPI-0610), a Small-Molecule Inhibitor of BET Proteins, in Patients with Relapsed or Refractory Lymphoma
Source: Cancer Res Commun. 2022 Aug 11;2(8):795–805. doi: 10.1158/2767-9764.CRC-22-0060 (PMC10010313; doi:10.1158/2767-9764.CRC-22-0060)
Supplement: Figure S3 — PK/PD Analysis of the Relationship Between Maximum Relative Change in Gene Expression and Pelabresib Exposure Metrics. [file crc-22-0060-s04.pptx]

## Slide 1
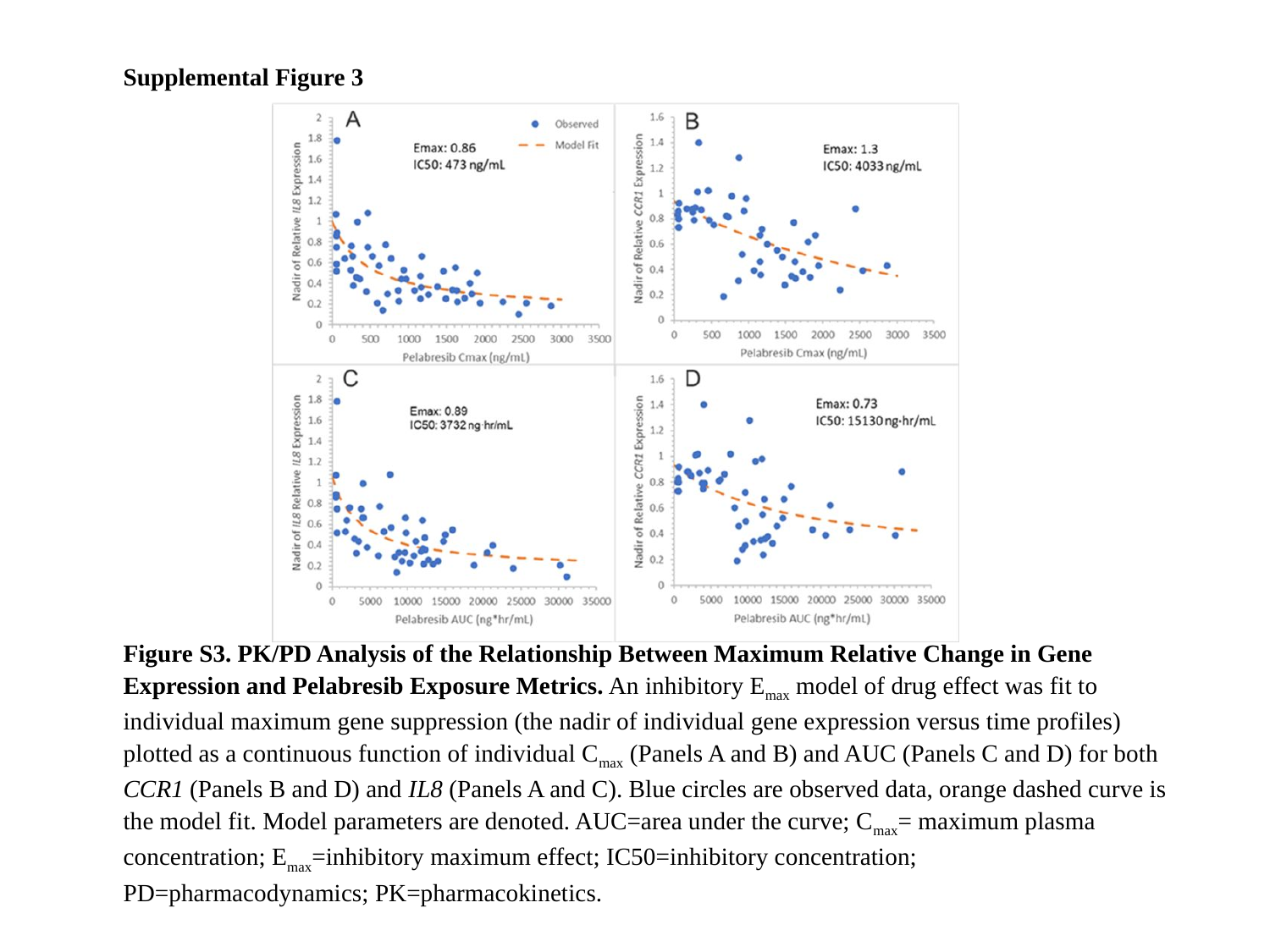

Supplemental Figure 3
Figure S3. PK/PD Analysis of the Relationship Between Maximum Relative Change in Gene Expression and Pelabresib Exposure Metrics. An inhibitory Emax model of drug effect was fit to individual maximum gene suppression (the nadir of individual gene expression versus time profiles) plotted as a continuous function of individual Cmax (Panels A and B) and AUC (Panels C and D) for both CCR1 (Panels B and D) and IL8 (Panels A and C). Blue circles are observed data, orange dashed curve is the model fit. Model parameters are denoted. AUC=area under the curve; Cmax= maximum plasma concentration; Emax=inhibitory maximum effect; IC50=inhibitory concentration; PD=pharmacodynamics; PK=pharmacokinetics.
